# Hidden Patterns of Anti-HLA Class I Alloreactivity Revealed Through Machine Learning

**DOI:** 10.3389/fimmu.2021.670956

**Published:** 2021-07-27

**Authors:** Angeliki G. Vittoraki, Asimina Fylaktou, Katerina Tarassi, Zafeiris Tsinaris, Alexandra Siorenta, George Ch. Petasis, Demetris Gerogiannis, Claudia Lehmann, Maryvonnick Carmagnat, Ilias Doxiadis, Aliki G. Iniotaki, Ioannis Theodorou

**Affiliations:** ^1^Immunology Department & National Tissue Typing Center, General Hospital of Athens “G. Gennimatas”, Athens, Greece; ^2^National Peripheral Histocompatibility Center, Immunology Department, Hippokration General Hospital, Thessaloniki, Greece; ^3^Immunology-Histocompatibility Department, “Evangelismos” General Hospital, Athens, Greece; ^4^Department of Computer Science & Engineering , University of Ioannina, Ioannina, Greece; ^5^Laboratory for Transplantation Immunology, Institute for Transfusion Medicine, University Hospital Leipzig, Leipzig, Germany; ^6^Laboratoire d’Immunologie, Hôpital St. Louis, Paris, France; ^7^Nephrology and Transplantation Unit, Medical School of Athens, Laikon Hospital, Athens, Greece; ^8^Centre d’Immunologie et des Maladies Infectieuses UPMC UMRS CR7-Inserm U1135-CNRS ERL, Paris, France

**Keywords:** machine learning, antigenic epitopes, alloimmune response, translational research, sensitization, bead array test, anti-HLA alloantibodies

## Abstract

Detection of alloreactive anti-HLA antibodies is a frequent and mandatory test before and after organ transplantation to determine the antigenic targets of the antibodies. Nowadays, this test involves the measurement of fluorescent signals generated through antibody–antigen reactions on multi-beads flow cytometers. In this study, in a cohort of 1,066 patients from one country, anti-HLA class I responses were analyzed on a panel of 98 different antigens. Knowing that the immune system responds typically to “shared” antigenic targets, we studied the clustering patterns of antibody responses against HLA class I antigens without any *a priori* hypothesis, applying two unsupervised machine learning approaches. At first, the principal component analysis (PCA) projections of intra-locus specific responses showed that anti-HLA-A and anti-HLA-C were the most distantly projected responses in the population with the anti-HLA-B responses to be projected between them. When PCA was applied on the responses against antigens belonging to a single locus, some already known groupings were confirmed while several new cross-reactive patterns of alloreactivity were detected. Anti-HLA-A responses projected through PCA suggested that three cross-reactive groups accounted for about 70% of the variance observed in the population, while anti-HLA-B responses were mainly characterized by a distinction between previously described Bw4 and Bw6 cross-reactive groups followed by several yet undocumented or poorly described ones. Furthermore, anti-HLA-C responses could be explained by two major cross-reactive groups completely overlapping with previously described C1 and C2 allelic groups. A second feature-based analysis of all antigenic specificities, projected as a dendrogram, generated a robust measure of allelic antigenic distances depicting bead-array defined cross reactive groups. Finally, amino acid combinations explaining major population specific cross-reactive groups were described. The interpretation of the results was based on the current knowledge of the antigenic targets of the antibodies as they have been characterized either experimentally or computationally and appear at the HLA epitope registry.

## Introduction

Antibody response against human leucocyte antigens (HLAs) is among the most studied immune parameters for patients on the waiting list and post-organ transplantation (Tx). In solid organ transplantation, a full HLA match between a donor and a recipient is the exception rather than the rule. Incompatible graft HLA may become targets of preformed before transplantation (Tx) antibodies but they may also activate anti-graft alloresponses post Tx leading to graft injury and rejection (1, 2).

Anti-HLA antibody responses usually have a broader spectrum in addition to immunogenic antigen, as they are directed against several HLA which are not presented by the graft or other pre-graft immunogenic sensitization events such as HLA of the father during pregnancy or blood donors HLA. This anti-HLA cross-reactivity can become a major problem which is especially harmful in the setting of a second transplantation.

The main theory put forward to explain cross-reactivity is that HLA molecules show antigenic similarities rendering an immune response against an “unseen” HLA allele. This property of immune responses against HLA antigens has been observed very early in the history of HLA discoveries with the first Cross Reactive Epitope Group (CREG) for HLA-B antigens describing this feature as early as 1963 (3). Cross-reactions are thought to be due to specific amino acid (aa) linear or conformational combinations designated as triplets and later eplets shared by different HLA alleles (4, 5). Some of these short aa sequences have been experimentally described, but others have been devised by indirect methods (6–8). Based on eplet, triplet or more recently simple electrochemical distances of HLA molecules between a donor and a recipient, several predictive algorithms have been proposed and are used in transplantation settings (9–11). Certainly, finding objective methods measuring these distances could be of significant importance to improve predictive algorithms based on donor–recipient HLA mismatches. The algorithms mentioned evaluate the probability of an anti-HLA response against the graft. Although these algorithms may give different results, they are all based on an antigenic distance between the HLA molecules of the donor and the recipient for the prediction of a harmful immune response (12).

Here, an alternative method to measure the HLA antigenic distance is proposed, by studying the humoral alloresponse products in the serum of the patient with unsupervised machine learning approaches. The method provides models reflecting antigenic similarities between products of the same but also different loci.

Previously, we analyzed anti-HLA class II responses with unsupervised machine learning algorithms and demonstrated that this type of analysis describes most of the known patterns of the anti-HLA class II response (13). In this study, antibody fluorescent intensities as measured on a Luminex platform for 98 different HLA class I antigens per patient were analyzed in a cohort of 1,066 patients coming from a single country. This approach can be considered as an objective methodology to be used for grouping and measuring of antigenic distances of HLA class I molecules.

The results of this study suggest that antigenicity of HLA molecules can be revisited automatically, without any *a priori* hypothesis from Luminex data, provided that a big number of responses are studied. Principal component analysis (PCA) (14) projections on several orthogonal plane of intra- and inter-locus specific responses revealed that the reactions show a strong grouping tendency indicating associated and distantly related responses in the population. A second algorithm based on the study of the complete repertoire provided from a Luminex based assay revealed hierarchical clustering of allele-specific immune responses that projected as a dendrogram and bring to light new correlations and in a way refine previous findings of allelic response antigenic clustering (15). The groups of responses that emerged from this analysis were identified as “hidden patterns” underlying the diversity of polyclonal antibody profiles seen in a large series of patients.

## Materials and Methods

### Study Group

All patients (n = 1066) with anti-HLA class I single antigen bead (SAB) test in the three major histocompatibility laboratories in Greece during years 2017 and 2018 have been included in the study. The patients were 49 ± 14 years old, and 56% of them were males. Patients were either awaiting a kidney transplantation (n = 660) or monitored after kidney transplantation (n = 406). Kidney transplant candidates were tested for antibodies every three months in accordance with the Greek legislation, while patients after transplantation were monitored annually for anti-HLA antibodies unless clinical indications suggested a more frequent check-up. Sera were tested for antibody identification by SAB analysis after a positive LSM screening test from One Lambda-OL (22801 Roscoe Blvd West Hills, USA) or after a known positive historical test. In addition, the most recent serum samples of patients before transplantation as well as samples of patients with clinical suspicion of rejection were also tested with SAB independently of the LSM result.

The protocol was approved by the Health Research and Ethical Board of “G. Gennimatas” Hospital of Athens, “Hippokration” Hospital of Thessaloniki and “Evangelismos” Hospital of Athens. Informed consent of the patients for the study was obtained in accordance with the Declaration of Helsinki.

### Antibody Detection and Identification

Sera were tested with the SAB anti-HLA class I antibody detection kit from OL on a Luminex 100 flow multicolor cytometer. In order to reduce lot-to-lot variability only results obtained from lots 10 and 11 of LABScreen SAB Class I kits were included in the analysis. Laboratory tests were performed according to the instructions of the manufacturer. All sera were stored at −20°C until testing. EDTA pretreatment was performed on all sera to prevent the prozone effect as previously described (16).

Raw mean fluorescence intensities (MFIs) of the samples were exported from the Fusion Analysis software database. Only one sample per patient was included in the study. In cases where there were more than one serum sample from the same patient, only the most recent one was taken into account.

### Descriptive Statistics

Single locus, as well as an additive three loci analyses, was used to project antigenic groups in this population on several orthogonal planes defined by PCA (14). In order to reinforce the unsupervised character of the PCA, we took into account raw MFI values for all antigens even when they were evaluated as negative when one uses cut-off values.

For the additive three loci model, we used as input four variables resulting from the summed raw MFI values for anti-HLA-A, HLA-B, and HLA-C responses for each patient as well as a summed MFI value of all beads. Before performing the PCA summed values were centered and standardized to avoid variability inherent to disparities of inter-bead fluorescence maxima and minima and the fact that the number of antigens studied is different for each locus. This standardization pre-processing step was also mandatory since the number of beads was different for each locus, producing thus different locus-specific scales.

For locus-specific analyses, all antigen-specific raw MFI values had been taken into account as independent variables. We also centered and standardized MFI values and limited the representations on the first three principal components.

PCA was performed in R with the FactoMiner and Factoextra packages (17, 18).

We also used a second algorithm, based on hierarchical clustering (19) of allele-specific immune responses, in this same population, and visualized on a 2D plane as phylogenetic trees (20).

Before performing the calculations, raw MFI values had been centered around zero and divided by the standard deviation. Raw MFI values from all patients and for each bead were then used as feature vectors, and their relative distances were used for clustering. For the experiments presented, we employed the widely used Minkowski distance followed by a Wald’s based bottom to top hierarchical clustering (hclust in R).

Spatial ordering of the different antigens was not based on pairwise Spearman’s linear correlations (as PCA does for defined plane of the two first eigenvectors) but on Minkowski’s distances between antigen-specific feature vectors, capturing thus potential non-linear correlations among antibody responses.

Amino acid sequences of the mature protein of relevant alleles were retrieved from the FTP Directory of IPD-IMGT/HLA database (https://www.ebi.ac.uk/ipd/imgt/hla/) and plotted with Jalview (21). Amino acid coloring was based on its hydrophobicity (22). The most hydrophobic residues were colored red, and the most hydrophilic ones were colored blue.

Information on HLA-A, -B and -C antibodies verified epitopes on the 98 Luminex alleles; the name of the eplets and the ElliPro score came from the HLA Epitope Registry (http://www.epregistry.com.br), version 3.0, ABC database, Luminex alleles (23).

## Results

### PCA Projections of the Overall Anti-HLA-A, -B, -C Immune Responses

**Figure 1:**
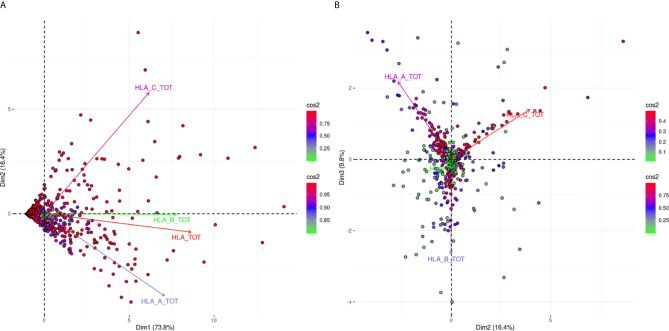
PCA biplot projections of locus specific and total (HLA-A, -B, -C loci) summed MFI values. Panel **(A)** (left part) Projects variables and individuals on the first PCA-defined plane, while Panel **(B)** (right part) represents the second PCA plane. The points represent projections of individual reactions and arrows of the different anti-HLA class I responses. The color vectors on the right side of both panels represent the cos2 value of the explained variance for variables and individuals respectively.

**Figure 1** is a biplot projection of a PCA of the sums of all MFI values for each HLA locus separately and the total MFI value from all three loci of each response. Arrows represent the different antigens considered as the variables of the PCA, while each dot represents an individual response.

**Figure 1A** shows the projections according to the delimited plane of the first two eigenvectors of PCA. This projection captures 90.2% of the total variance of anti-HLA class I immune response seen at a population level according to the summing of all MFI values for all the antigens of the same locus. The major characteristic of this projection was that all arrows (variables) pointed to the right. This suggested that immune responses of the population against HLA class I molecules were usually directed against all three loci. However, the anti-HLA-A and anti-HLA-C responses were almost orthogonal while HLA-B and HLA-TOTAL best explaining arrows were almost co-linear to the first eigenvector. Orthogonality between HLA-C and HLA-A MFI sums corresponding arrows was characteristic of distantly related responses showing that the two loci specific responses were weakly correlated.

For the interpretation of the orthogonality of the anti-HLA-A and anti-HLA-C responses, the hitherto known intra-locus “shared” antibody verified epitopes were sought (23). Only five “shared” between HLA-A and HLA-C intra-locus epitopes had been considered, which constituted 4.3% of the known HLA-A allele epitopes and 6.3% of the known epitopes for HLA-C. On the contrary, 55 intra-locus “shared” epitopes had been considered between HLA-B and HLA-A alleles (N = 24) and HLA-B and HLA-C alleles (N = 31) which constituted 18.6 and 24% of the total HLA-B epitopes respectively. This observation may justify the projection of the HLA-B responses as co-linear to the first eigenvector.

**Figure 1B** shows a biplot PCA projection according to the second and third eigenvectors. This projection explained the additional 9.8% of the variance and was characterized by arrows (variables) with almost 120° relative angles. Since these arrows are the best fitting lines of each variable, this finding proposes that only 9.8% of additional variance can be explained by an anti-HLA-A or HLA-B or HLA-C specific response. This second biplot put many “green” individuals (individuals weakly contributing to the overall variance) at the center of the biplot. On the contrary “red” individuals (strongly contributing to the overall variance) were almost very close to the arrow representing the locus-specific antigenic response further supporting the fact that mono locus-specific responses are rare.

### PCA Projections of the Anti-HLA-A Immune Response

**Figure 2:**
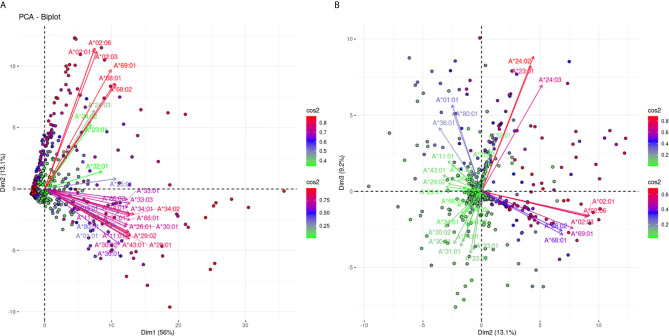
PCA biplot projections of the anti-HLA-A antibody response. Panel **(A)** on the left represents the first and second principal components defined plane, while Panel **(B)** on the right represents the defined plane of the second and third principal components. The points represent projections of individual reactions, and arrows represent the different anti-HLA-A responses. The color vectors on the right side of both panels represent the cos2 value of the explained variance for variables and individuals respectively.

The projections for the responses against 31 HLA-A alleles are shown in **Figure 2**. **Figure 2A** shows the projections according to the defined plane of the first two eigenvectors and captures 69.1% of the variance of the anti-HLA-A immune response, while **Figure 2B** captures another 9.2% of the variance reaching 78.3% of the total variance. In **Figure 2A** we can observe roughly three groups of variable clustering. Two distant and well-explained anti-HLA-A allele-specific responses showing an almost orthogonal pattern (long red arrows) visible on this projection. It appears that responses against the A*02:01, *02:03,*02:06,*68:01, *68:02, and *69:01 (bead-array defined group 1), known as A2C1 or 2C (A2) CREG occupied the upper quadrant, while the beam of long red arrows corresponding to anti-HLA-A *01:01, *03:01, *11:01, *11:02, *26:01, *29:01, *29:02, *30:01,*30:02, *31:01, *33:01, *33:03, *34:01, *34:02, *36:01, *43:01, *66:01, *66:02, *74:01, and *80:01 reactions (bead-array defined group 2) occupied the lower quadrant of the biplot, defining a second major group. This finding suggests that in the Greek population there are two main types of unrelated responses against HLA-A proteins “captured” in the first PCA biplot. The third group of clustering consisting of the arrows located at the area between the two beams of long red arrows, represented reactions against HLA-A *23:01, *24:02, *24:03, *25:01, and *32:01 (bead-array defined group 3) “weakly” explained on this biplot (shorter green and blue arrows). Worth noting, group 3 alleles belonging to the HLA Bw4 CREG (24) and suggesting that HLA Bw4 was associated with structural variation of HLA-A molecules was another attribute “captured” by PCA, though this clustering was mostly characterized by weak variance explanation in the first plane PCA projection.

**Figure 2B** confirms the negative correlation between the two major groups of **Figure 2A**. In addition, it shows clear orthogonality between the group 1 alleles and the “poorly” explained third group on Panel 2A. We further sought to explore whether the two most uncorrelated groups of anti-HLA-A responses seen through this PCA model are associated with shared structural polymorphisms.

**Figure 3:**
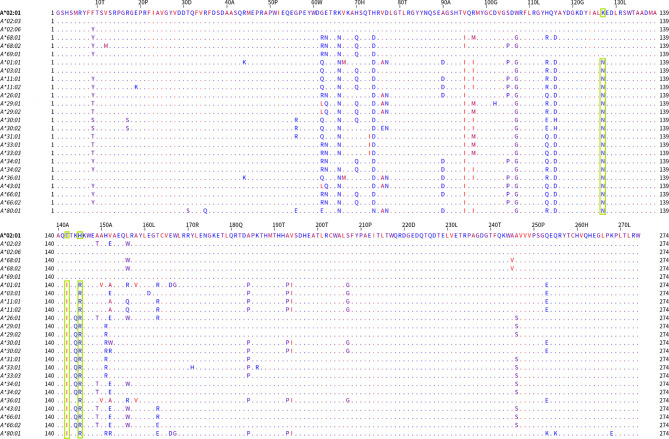
Mature protein sequence alignments for PCA defined groups of HLA-A antigens. Residues are colored according to the hydrophobicity scale of Kyte, J., and Doolittle, R.F. This scale can be used for identifying surface-exposed regions. The most hydrophobic residues are colored red, and the most hydrophilic ones are colored blue. Changes in the patterns of amino acid conservation between group 1 (A*02:01, A*02:03, A*02:06, A*68:01, A*69:02) and group 2 (A*01:01, A*03:01, A*11:02, A*26:01, A*29:01, A*29:02, A*30:01, A*30:02, A*31:01, A*33:01, A*33:03, A*34:01, A*34:02, A*36:01, A*43:01, A*66:01, A*66:02, A*74:01, A*80:01) are highlighted in green rectangles. Described eplet 144TKH is the overall distinctive factor between the groups that PCA projects.

In **Figure 3**, the alignment of the group 1 belonging alleles (upper part of the alignment graph) and the group 2 (lower part of the alignment graph) alleles are shown. The residues were colored according to their hydrophobicity as proposed by Kyte J and Doolittle R (22). When the members of these two bead-array defined groups were aligned, they differed at position 127 (K/N) and positions 142 and 145 (T, H/I, R). Two of these positions corresponded to the antibody verified eplets 144 TKH and 145KHA. Two other noteworthy structural variants were located at positions 95 (V/I) and 107 (W/G), showing that at those positions the A*69:01 allele (the most proximal allele to A*02 alleles in the PCA biplot) was identical to the A*02 alleles, while all other alleles shared a different aa at the respective positions.

The PCA projections of the HLA-A responses into three distinct groups, reaching 78.3% of the total variance, suggest that the antibodies recognize “non-shared” between the groups polymorphic regions. According to this hypothesis, a table was formed with 45 antibody targets—eplets, as they appear at the HLA epitope registry: eight eplets were expressed on group 1 alleles, 29 eplets were expressed on group 2 alleles, and eight eplets were expressed on group 3 alleles. The co-expression on other locus alleles was also indicated. Six out of eight polymorphic regions expressed on A*23, *24, *25, *32 group 3 alleles were shared with HLA-B alleles (**Table 1**). The surface expression of the corresponding eplets had been drawn either from the Epitope registry or calculated by a convolutional and long short-term memory neural networks trained on solved protein structures NetSurfP-2.0 algorithm (25).

**Table 1:** Antibody verified eplets for the three PCA defined HLA-A groups with the one lambda bead array assay.

HLA-A Groups	Eplets within the group	Alleles	Co-expressed on other loci alleles	ElliPro score	RSA score
1st Group					
A*02:01, A02:03, A*02:06, A*68:01, A*68:02, A*69:01	12M	A*68:02	B locus	H	0,19
	62GE	A*02 whole	B locus	INT	0,4
	62GK	A*02 whole		INT	0,4
	95V	A*02 whole, A*69		L	0,1
	144TKH	whole group		H	0,6
	145HT	A*02:03		H	0,6
	145KHA	whole group		H	0,6
	245V	A*68		INT	0,04
2nd Group					
A*01:01, A*03:01, A*11:01, A*11:02, A*26:01, A*29:01, A*29:02, A*30:01, A*30:02, A*31:01, A*33:01, A*33:03, A*34:01, A*34:02, A*36:01, A*43:01, A*66:01, A*66:02, A*74:01, A*80:01	9T	A*29,*31,*33		VL	0,08
	17S	A*30		H	0,62
	19K	A*11:02		H	0,45
	35Q	A*80	C locus	L	0,34
	44KM	A*01,*36		H	0,52
	56E	A*80		H	0,52
	56R	A*30,*31		H	0,45
	62LQ	A*29,*43		INT	0,47
	66NM	A*01,*36	B locus	L	0,45
	73ID	A*31,*33		INT	0,41
	76ANT	A*01,*26,*29,*36,*43,*80		H	0,51
	76EG	A*30:02		H	0,64
	76ET	A*30:02	B locus	H	0,64
	77NGT	A*01,*26,*29,*30:02,*36,*43,*80		H	0,3
	97I	A*01,*03,*11,*30,*34,*36,*80		VL	0,07
	102H	A*29:01		VL	0,37
	151AHA	A*11		H	0,74
	152A	A*01,*11,*36	C locus	H	0,25/0,23/0,24
	152HA	A*01,*11,*36		H	0,4
	152RR	A*30,*80		H	0,21/0,41/0,4
	152W	A*30:01		H	0,21
	156R	A*01,*36	B and C loci	INT	0,18
	161D	A*03:01		H	0,43
	163E	A*66:02,*80	B and C loci	INT	0,28/0,33
	163EW	A*66:02	B and C loci	INT	0,28
	163RG	A*01:01		H	0,31
	170RH	A*33:01	B locus	H	0,43
	186R	A*33:01		H	0,62
	275EL	A*01,*03,*11, *30,*36		H	0,9
3rd Group					
A*23:01, A*24:02, A*24:03, A*25:01, A*32:01	65GK	A*23,*24		INT	0,4
	76ES	A*25,*32	B locus	H	0,55
	76ESI	A*25,*32		H	0,55
	77S	A*25,*32	B and C (C1) loci	INT	0,31/0,26
	80I	whole group	B locus	H	0,37/0,38/0,45/0,41
	81ALR	whole group	B locus	H	0,1/0,1/0,2/0,13
	82LR	whole group	Bw4 alleles	H	0,2/0,2/0,3/0,2
	99F	A*23,*24	B and C loci	VL	0,1

Antibody targets -eplets of the HLA epitope registry and their expression calculated either with ElliPro or NetSurf are indicated. The RSA score relates to the NetSurfP's Relative Solvent Accessibility and aminoacid with values greater than 0.25 are considered as surface expressed. The * is part of the HLA nomenclature (separator). Τhe symbol * identifies antigens in the form of alleles (4 digits). When used in 2 digit antigen means that it refers to all alleles studied.

### PCA Projections of the Anti-HLA-B Immune Response

**Figure 4:**
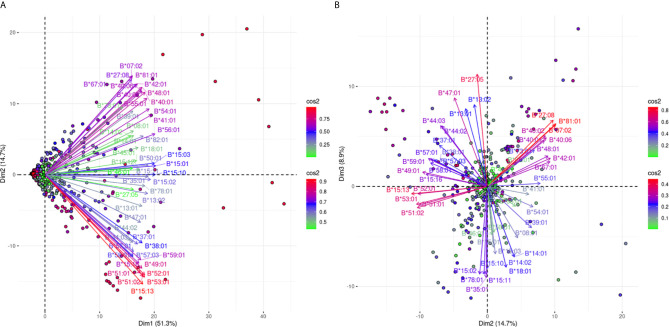
PCA biplot projections of the anti-HLA-β antibody response. Panel **(A)** on the left represents the defined plane of the first and second principal components while Panel **(B)** on the right represents the second and third principal components defined plane. The points represent projections of individual reactions and arrows the different anti-HLA-B responses. The color vectors on the right side of both panels represent the cos2 value of the explained variance for variables and individuals respectively.

**Figure 4A** shows an analogous PCA projection for the responses against 51 HLA-B alleles and explains 66% of the variance on the first best explaining PCA biplot. The main characteristic of this projection is that arrows corresponding to the different reactions were not densely grouped, suggesting that the anti-HLA-B alleles consisted of many different cross-reactive groups. However, there is a clear distinction of anti-Bw4 and anti-Bw6 reactions occupying the lower and upper quadrants of the PCA model respectively. Furthermore, some characteristic orthogonalities were made obvious, such as those representing reactivity against B*07:02, B*27:08, and B*81:01 (Bw6 CREG alleles) and B*15:13, B*51:01, B*51:02, B*52:01, and B*53:01 (Bw4 CREG alleles) which were the most distantly related.

In order to increase the explained variance of HLA-B specific responses we also projected the 2nd and 3rd eigenvector limited planes (**Figure 4B**). Another 8.9% of the overall variance can be observed in this graph, achieving a total of 74.9% of the overall variance. Orthogonality within the Bw4 and Bw6 belonging antigens was still the main characteristic through this second projection. Moreover, subgroups within anti-Bw4 (on the left) and anti-Bw6 (on the right) reactions were formed. Two negatively correlated cross-reactive subgroups within anti-Bw4 reactions that included anti-B*27:05 and B*47:01 reactions (upper left quarter) and B*15:13, B*51:01, B*51:02, B*52:01 and B*53:01 reactions (lower left quarter) were defined. Accordingly, two negatively correlated cross-reactive subgroups within anti-Bw6 reactions that include anti-B*07:02, B*27:08 and B*81:01 reactions (upper right quarter) and anti-B*15:02, B*15:10, B*15:11, B*35:01, and B*78:01 reactions (lower right quarter) were observed.

Investigating whether some cross-reactive groups that are visible within the two PCA projections can be such due to strong antigenic similarities of the corresponding alleles, we explored sequence similarities according to the correlation patterns observable on the first and second PCAs delimited planes which clearly separate subgroups of the anti-Bw4 and anti-Bw6 reactions.

**Figure 5:**
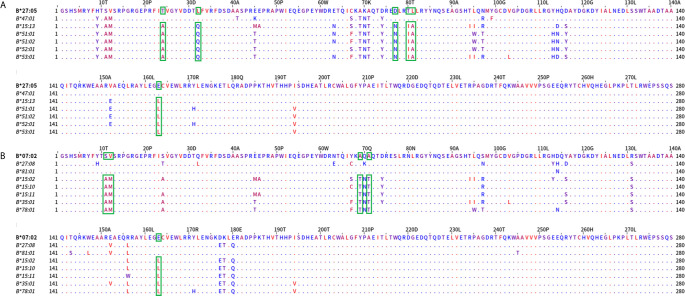
Mature protein sequence alignments of distantly PCA defined groups of HLA-B antigens. Upper panel **(A)** represents the most distantly related alleles grouped according to PCA and Bw4 belonging alleles. Residues are colored according to the hydrophobicity scale of Kyte, J., and Doolittle, R.F. This scale can be used for identifying surface-exposed regions. The most hydrophobic residues are colored red, and the most hydrophilic ones are colored blue. Changes in the patterns of amino acid conservation between group 1 (B*27:05, B*47:01) and group 2 (B*15:13, B*51:01, B*51:02, B*52:01 and B*53:01) are enclosed in a green rectangle. Lower panel **(B)** represents the most distantly related alleles grouped according to PCA and the Bw6 groups. Changes in the patterns of amino acid conservation between group 1 (B*07:02, B*27:08, B*81:01) and group 2 (B*15:02, B*15:10, B*15:11, B*35:01, B*78:01) are enclosed in a green rectangle.

We first performed an analysis on the Bw4 most uncorrelated reactions (**Figure 5A**) represented by B*27:05 and B*47:01 forming the first group *versus* B*15:13, B*51:01, B*51:02, B*52:01, and B*53:01 forming the second group; the analysis showed aa differences at positions 24 (T/A), 32 (L/Q), 77, 80, 81 (D,T,L/N,I,A), and 163 (E/L). They correspond to eplets 24T (very low ElliPro score), 32L (very low ElliPro score), 77D, and 77N (intermediate ElliPro score), 80T, 80TL, 80I (high Ellipro score), 163L, 163E, 163EW (all three with intermediate ElliPro score) and 163LE, 163LW (both with high ElliPro score). Only 80I, 163EW, and 163LW eplets were antibody verified.

Then we compared the most distant Bw6 specificities. As shown in **Figure 5B**, the orthogonality between the B*07:02, B*27:08, and B*81:01 cross-reactive group and the uncorrelated B*15:02, B*15:10, B*15:11, B*35:01, and B*78:01 group was associated with aa differences between the two groups at positions 11, 12 (S,V/A,M), 69, 71 (A,A/T,T), and 163 (E/L). Eplets described in these positions included 12M (high ElliPro score), 69AA (intermediate ElliPro score), and 69TNT (intermediate ElliPro score), 163L, (intermediate ElliPro score), 163LE and 163LW (both with high ElliPro score), 163E and 163EW (both with intermediate ElliPro score). Only 69AA, 69TNT, 163LW, and 163EW eplets were antibody verified.

### PCA Projections of the Anti-HLA-C Immune Response

**Figure 6:**
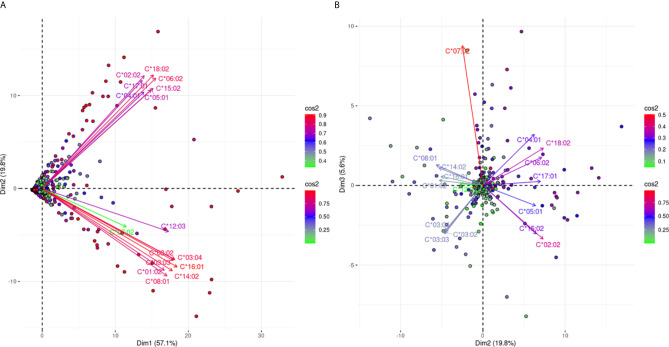
PCA biplots of the anti-HLA-C immune response. Panel **(A)** on the left represents the defined plane of the first and second principal components while Panel **(B)** on the right represents the defined plane of the second and third principal components. The points represent projections of individual reactions and arrows the different anti-HLA-C antigen-specific responses. The color vectors on the right side of both panels represent the cos2 value of the explained variance for variables and individuals respectively.

**Figure 6** projects the antibody responses against 16 different HLA-C alleles. **Figure 6A** explains 76.9% of the total variance and shows a clear bi-grouping of two beams of arrows. The two groups were still negatively correlated in **Figure 6B** which captures an additional 5.6% of the variance, reaching 82.5% of the total anti-HLA-C responses. However, there was a slight distinction for anti-HLA-C*07:02 responses that seemed to be weakly explained on the first plane. In **Figure 6B** the anti-HLA-C*07:02 response remained in the same group as before but showed clear orthogonality with the responses against the HLA-C*03 alleles. These observations potentially represented a most distantly related antigenic structure of HLA-C*07:02 within the HLA-C alleles.

**Figure 7:**
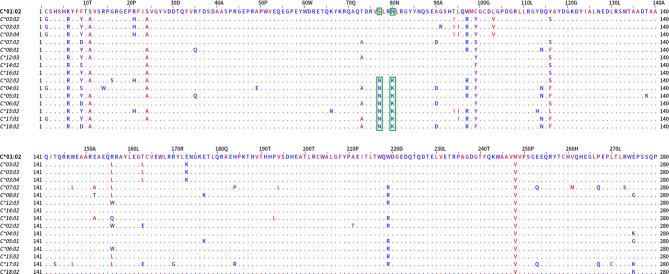
Mature protein sequence alignments of PCA defined groups of HLA-C antigens. Residues are colored according to the hydrophobicity scale of Kyte, J., and Doolittle, R.F. This scale can be used for identifying surface-exposed regions. The most hydrophobic residues are colored red, and the most hydrophilic ones are colored blue. Changes in the patterns of amino acid conservation between group 1 (C*01:02, C*03:02, C*03:03, C*03:04, C*07:02, C*08:01, C*12:03, C*14:02, C*16:01) and group 2 (C*02:02, C*04:01, C*05:01, C*06:02, C*15:02, C*17:01, C*18:02) are enclosed in a green rectangle. Described eplets 76VS, 77S, 80N for group 1 *vs* eplets 77N and 80K for group 2 are the distinctive factors between the groups that PCA projects.

When the sequences of the two allele groups were aligned, two dimorphisms, one at position 77 (S/N) and a second at position 80 (N/K) differed between these two antigenic groups (**Figure 7**).

Again, the projections of the HLA-C responses into two distinct groups, reaching 82.5% of the total variance, suggest that the polymorphisms recognized from the antibodies should be expressed exclusively on the alleles of each group. According to this hypothesis a table was formed with the 35 antibody targets—eplets as they appeared at the HLA epitope registry: 20 eplets were expressed on C1 alleles, and 15 eplets were expressed on C2 alleles. HLA-C*07:02 was not included in the analysis. The expression on other loci/alleles is also indicated (**Table 2**).

**Table 2:** Antibody verified eplets for the two PCA defined HLA-C groups with the One Lambda bead array assay.

HLA-C Groups	Eplets within the group	Alleles	Co-Expressed on other loci alleles	ElliPro score	RSA
C1 Group					
C*01:02, C*03:02, C*03:03, C*03:04, C*08:01, C*12:03, C*14:02, C*16:01	9F	C*01:02	A locus	VL	0,17
	73AS	C*12:03		INT	0,56
	73TVS	all ex C*12:03	B*46:01	H	0,55
	76VRN	whole group	B*46,*73	H	0,35
	76VS	whole group	B*46	H	0,35
	77S	whole group	A and B loci	INT	0,48
	77SRN	whole group	B locus	H	0,48
	80N	whole group	Bw6 allels	H	0,55
	91R	C*03:03		H	0,6
	116Y	C*01:02,*03:03,*03:04	A and B loci	VL	0,21
	152A	C*16:01	A locus	H	0,71
	152RA	C*16:01		H	0,71
	152T	C*08:01		H	0,67
	156QA	C*16:01	A locus	INT	0,56
	163L	C*03 whole group	B locus	INT	0,43
	163LE	C*03 whole group	B locus	H	0,43
	163LW	C*03 whole group	B locus	H	0,43
	173K	C*03 whole group		H	0,2
	193LV	C*16:01		H	0,56
	248M	C*01:02		H	0,02
C2 Group					
C*02:02, C*04:01, C*05:01, C*06:02, C*15:02, C*17:01, C*18:02	14W	C*04:01		H	0,13
	16S	C*02:02		H	0,43
	73AN	C*04,*17,*06,*18		INT	0,55/0,56/0,55/0,56
	77N	whole group	A and B loci	INT	0,47
	80K	whole group		H	0,55
	113HD	C*15:02	B locus	VL	0,07
	116L	C*15:02	B locus	VL	0,22
	138K	C*05:01,*08:02		H	0,41/0,41
	143S	C*17:01	B locus	H	0,4
	163E	C*17:01,*02:02	A and B loci	INT	0,43/0,45
	163EW	C*17:01,*02:02	A and B loci	INT	0,43/0,45
	184R	C*17:01		INT	0,5
	211T	C*02:02		VL	0,05
	270C	C*17:01	B*73:01	H	0,51
	275K	C*04:01,*17:01, *18:02	B*73:01	H	N/A

Antibody targets -eplets of the HLA epitope registry and their expression calculated either with ElliPro or NetSurf are indicated. The RSA score relates to the NetSurfP's Relative Solvent Accessibility and aminoacid with values greater than 0.25 are considered as surface expressed. The * is part of the HLA nomenclature (separator). Τhe symbol * identifies antigens in the form of alleles (4 digits). When used in 2 digit antigen means that it refers to all alleles studied.

### Phylogenetic Analysis of Anti-HLA Class I Immune Responses

**Figure 8:**
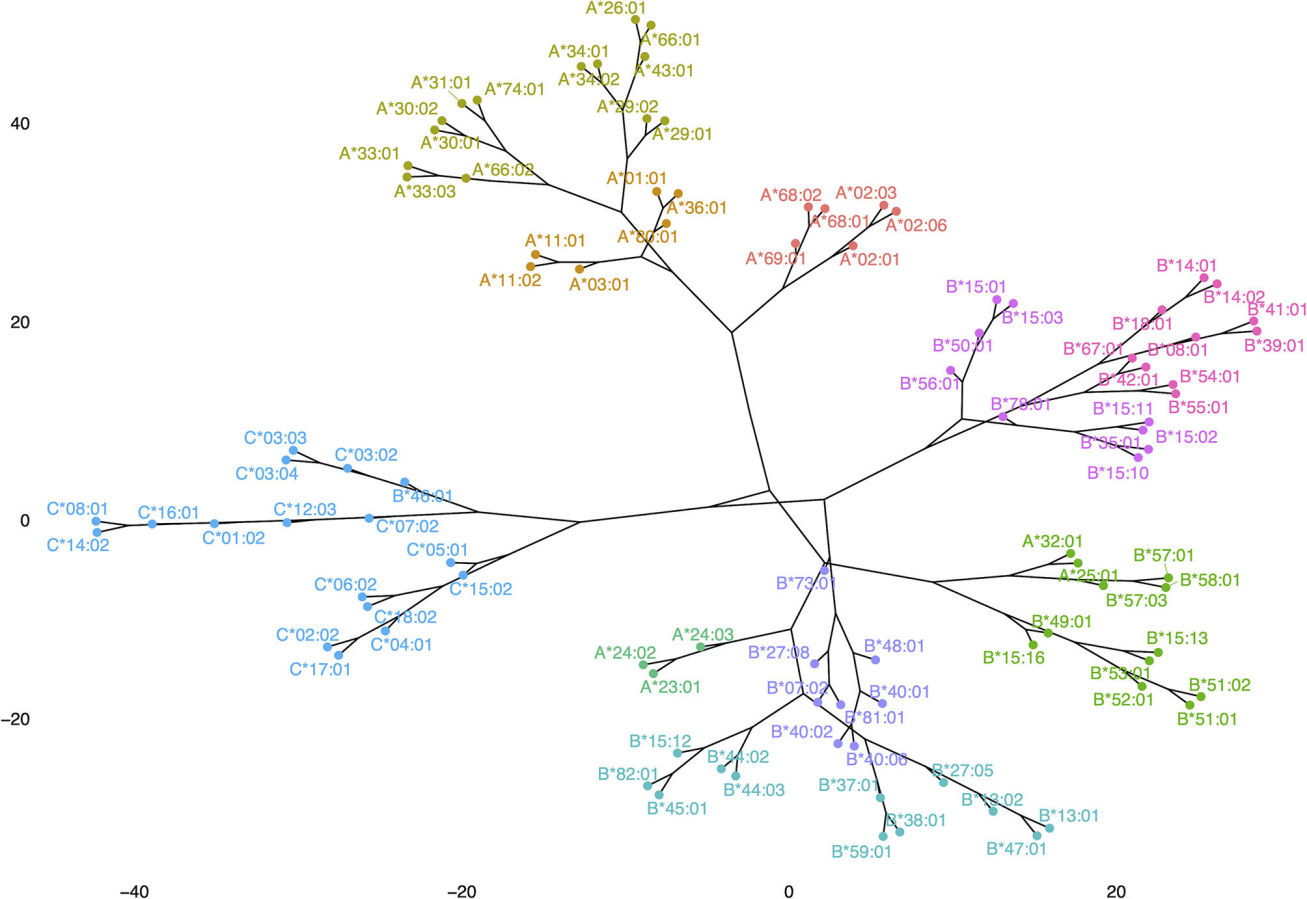
Representation of anti-HLA class I immune responses as a dendrogram produced for Minkowski calculated distances of antigen-specific feature vectors.

**Figure 8** shows a dendrogram where the Minkowski metric was used to calculate the antigenic distances of antigen specific feature vectors. These analyses brought to light new correlations and in a way refined previous findings of allelic response clustering, though based on the study of the complete repertoire provided from a Luminex based assay. This type of “species-like” representation of anti-HLA class I immune responses follows most of the main known facts about cross-reactivity of anti-HLA class I responses. Indeed, with almost no exceptions, antigens encoded by the different loci were within the same phylogenetic branch with few exceptions. Among these exceptions were responses against B*46:01 which is antigenically related to HLA-C responses (26) and anti-HLA-A*23, A*24, A*25, and A*32 responses which are antigenically related to HLA-B responses whatever the distancing method used.

## Discussion

Knowledge about cross-reactivity of anti-HLA responses has been gained over several years usually in the setting of solid organ transplantation and has provided a rationale for common observations such as the development of antibodies against non-donor antigens which are shared with the polymorphic regions of the donor antigens interacting with antibodies. These have been defined as eplets and represent a part of a B cell epitope.

Experimental definition of eplets can be of great value to improve risk stratification tools and hopefully further push to the direction of improved computational tools and their potential use as prognostic markers. Eplets were defined experimentally after adsorption of poly-specific antibodies on appropriate targets (homozygote cell line or recombinant HLA molecule) followed by an elution step. The eluate was then tested for reactivity on all other HLA molecules followed by the definition of amino acid similarities between the recognized antigens which defined the eplet (27). Therefore, the definition of eplets depends on the sera tested and several eplets could be ignored depending on the selection criteria for the tested sera. However, a high number of incompatible eplets on donor antigens increase the likelihood that some of these epitopes will be immunogenic, increasing the risk of developing donor-specific antibodies (12). Hence, Eurotransplant has used an eplet based allocation scheme for highly sensitized patients with excellent results (28, 29). This led to the idea that HLA eplet mismatch differences or other HLA molecular mismatch (mMM) scores are promising approaches for risk stratification although the definition of cut-off values defining a positive reaction is still an object of debate (30). Therefore, experimental definition of eplets can be of great value to improve these risk stratification tools and hopefully further push to the direction of improved computational tools and their potential use as prognostic markers (31). Moreover, there is no acceptable score of the immunogenic value of each eplet mismatch.

In the present study a method of “reading” the products of the humoral alloresponse in terms of their antigenic targets is proposed. Modeling the immune response with dimensionality reduction approaches produced very simple but robust unsupervised views of cross-reactivity, and thus an indirect eplet definition, as seen on Luminex analyzed immune responses. We preferred using unsupervised dimensionality reduction algorithms in order to let the data “speak” without any *a priori* immunological hypothesis. For this reason, we did not follow the instructions provided by the manufacturers to use arbitrary cut-off values to determine positive and negative reactions. The interpretation of the results was based on the current knowledge of the antigenic targets of the antibodies as they have been characterized experimentally or computationally and as they appear at the HLA epitope registry.

Previously, we undertook a machine learning approach in a homogeneous cohort coming from a single country in order to “revisit” cross-reactivities observed for anti-HLA class II responses in patients with anti-HLA class II antibodies either before or after renal transplantation (13). Now, we reasoned that extracting this type of information from an equivalent HLA class I specific data table could be also useful for the generation of concepts that could be helpful for bead-array eplet definition and give indirect information about antigenic distances of HLA alleles that could be useful for patient monitoring.

The first analysis presented in this manuscript showed that 90% of the variance of the immune response seen as cumulative MFI values for each locus can be captured on a single plane PCA biplot. This finding suggests that using the PCA as a dimensionality reduction algorithm is highly suitable for the analysis in question.

Furthermore, other indirect conclusions can be easily drawn from this biplot. Indeed, the fact that anti-HLA-A and anti-HLA-C responses show an almost orthogonal pattern suggests that these responses are weakly correlated at a population level and thus the antigenic distance between the products of these two loci are the biggest among the products of class I loci analyzed nowadays on Luminex devices. Furthermore, when we performed the same type of PCA with single bead fluorescence measures we also observed a similar orthogonal pattern between the alleles encoded by A and C loci although responses against 47 different alleles were analyzed (data not shown). These findings suggest that shared eplets among these two loci do not play an important role when seen from a population perspective. This is also in accordance with the number of common eplets published in the epitope registry.

On the other hand, total anti-HLA-B and total anti-HLA class I responses are colinear and lie along the first eigenvector of the PCA biplot thus suggesting that the major variance observed in anti-HLA class I response comes from anti-HLA-B response and has a strong correlation with the total anti-HLA class I response. The indirect message of this finding is that an increase in anti-HLA-B response correlates with an increase in total anti-HLA class I response and is thus strongly correlated with a high sensitization status. This finding is also in accordance with the number of proposed eplets in the epitope registry but also with the high degree of polymorphism of the HLA-B locus (23). The PCA decomposition of the antibody response highlights the polyclonal nature the response.

Furthermore, when we looked at the PCA biplot defined by the second and third eigenvectors of the covariance matrix, we observed a clear pattern of three 120° angled arrows suggesting that locus-specific reactions are rare when seen from a population perspective.

When all allele-specific MFIs for HLA-A locus on a PCA biplot are projected, 69.1% of the variance is captured on a single plane. Again, this fact argues that PCA is suitable for modeling the anti-HLA-A response. However, reactions against all antigenic specificities are not well captured on this single biplot. Indeed, anti-HLA-A2 and HLA-A28 bead-array defined group 1 responses are well represented on this biplot and almost orthogonal to group 2 allele responses which are also very well represented. Indirectly, this finding highlights that the anti-HLA-A response is more frequently directed against two major loosely bead-array defined groups of eplets.

The most interesting pattern of this projection is that there is almost no correlation between these two types of response suggesting that two major but independent types of immune responses are observable in the Greek population. A similar result is observed with a cohort analyzed with an Immucor bead-array (**Supplementary Figure 1A**).

There have been described 115 polymorphic residues accessible to antibodies on the 31 HLA-A alleles that were tested for antibodies in this study (23). According to the projections of the anti-A responses on PCA biplots, 69.1% of the response variance is explained by a strong clustering tendency, associated with only 45 of these polymorphisms. However according to these, only the antibody verified eplets 144TKH and 145KHA, which were found to be different between the two major bead array defined allelic groups, are HLA-A specific suggesting that their concomitant presence or absence associates with the two major groups of observable clusters in the Greek population. The fact that both eplets have amino acids which are predicted as being on the surface of the molecules suggests that a PCA analyzed anti-HLA-A response in a large population highlights the most important cluster associated with antibody verified eplets, but also may give experimental credit for eplets found only through computational approaches. In addition, the clear orthogonality observed in **Figure 2B** between the anti-A2C1 CREG allele responses and the responses against the HLA-A23, A24, A25, and A32 third group alleles, associates with the polymorphisms at position 82, 83 (RG/LR). These polymorphisms correspond to the 82LR eplet (high ElliPro score and antibody verified), expressed on the HLA- A23, A24, A25, A32, and all Bw4 alleles.

HLA-B specific responses show a much more complex pattern and the first plane PCA projection captures 66% of the variance. Indeed, the arrows representing the different antigens analyzed through this Luminex assay are more loosely grouped, though Bw4 associated antigens (variables) are grouped below the 1st eigenvector and Bw6 associated antigens are grouped above. This finding underlines the fact that the Bw4 and Bw6 CREG distinction of HLA-B response is the main feature clearly seen on Luminex-based assays. A similar result is observed with a cohort analyzed with an Immucor bead-array (**Supplementary Figure 1B**).

In order to capture at least 70% of the variance per locus, we also used a second plane projection by adding another 8.9% of explained variance for the B locus. Again, the Bw4 and Bw6 variable grouping is preserved. Some other characteristics of variable co-linearity are also noteworthy if the projections of the two planes are combined. Indeed, B*07:02, B*27:08, and B*81:01 (Bw6 alleles) specific arrows remain grouped whatever the plane is; thus, these antigens belong to one major bead-array defined group observed in the Greek population. Another bead-array defined group is composed of B*15:13, B*51:01, B*51:02, B*52:01, and B*53:01 (Bw4 alleles). These two groups are represented by two beams of arrows orthogonal to each other on the first PCA plane, and although still grouped, they are pointing in opposite directions on the second plane. Taken together these data suggest that antibody responses against these two groups are uncorrelated when one looks at the first PCA plane and for a minority of patients are negatively correlated (second PCA plane). Although this is not really surprising, it is interesting to note that these two Bw4 and Bw6 belonging groups represent the most distantly related and very well explained antibody responses against the B locus.

The second plane variance decomposition adds supplementary information represented as angular distances between the arrows representing each antigen related variable of the Bw4 CREG. Indeed, the B*27:05 and B*47:01 arrows are still grouped and almost orthogonal to the most distantly related B*15:13, B*51:01, B*51:02, B*52:01, and B*53:01 group. This suggests that these two groups show the maximum antigenic distance within the Bw4 CREG.

Moreover, on the second plane PCA biplot for the Bw6 CREG, a B*07:02, B*27:08, and B*81:01 bead-array defined sub-CREG shows a negative correlation with a bead-array defined sub-CREG formed by B*15:02, B*15:10, B*15:11, B*35:01, and B*78:01. One can thus argue that aa differences between distantly related groups may be seen as a data-based approach to define eplets with a strong impact on the population. Similarly, aa which differs between the alleles of each “tightly packed” group points to aa changes defining eplets with a limited “weight” in the studied population.

We then tried to see whether sequential alignments of the most distantly related Bw4 and Bw6 belonging alleles provide additional information about eplet weights.

We first performed these alignments for the most antigenically distant Bw4 alleles, and we found that only three (80I and 163EW and 163LW) of the eplets found to be different between these Bw4 alleles have been antibody verified. This fact suggests that several computationally defined eplets associate with a frequent cluster of an immune response and are specific of an antigenically similar PCA defined allelic group of foremost importance for the studied population.

For the most distantly related Bw6 aa differences at positions 11, 12 (SV/AM), 69, 71 (AA/TT), and 163 (E/L) between these sub-Bw6 groups are found and corresponded to eight eplets. Even though only three of the eight eplets have been experimentally verified, again a group of five computationally defined eplets associate with a frequent cluster of an immune response.

Taken into consideration these data, it is suggested that a sequential analysis of PCA either proximal or distant allelic groups may give new information about the importance (weight) of computationally derived eplets needing special attention. This might be important for a better understanding of anti-HLA-B responses where many computationally eplets are described.

Anti-HLA-C responses that are divided into two groups occupying the upper and lower quadrants of the PCA biplot, respectively represent 76.9% of the variance of the antibody response in the Greek population. Interestingly, the two groups of antibodies recognize the two groups of HLA-C alleles that express either the C1 or the C2 epitope. Both epitopes are known as the dominant inhibitory KIR ligands (32). These epitopes are characterized by different aas at position 80, where the presence of an asparagine (N) defines the C1 group while the presence of a lysine (K) defines the C2 group (33). There have been described 77 polymorphic residues on the 15 HLA-C alleles (excluding C*07:02) that were used as antibody targets in the Luminex assay. According to the projections of the anti-C responses by PCA, 82.5% of the response variance is explained by a strong clustering tendency associated with only 35 of the known polymorphisms. A similar pattern of anti-HLA-C responses is seen in a French and a German population (M. Carmagnat and C. Lehmann personal communications). Moreover, a similar pattern was observed in a cohort of patients analyzed on an Immucor bead array (**Supplementary Figure 1C**). This relatively tight biphasic pattern is surprising, and one should be cautious about potential confounding factors such as the presence of different proportions of denatured HLA molecules on the bead array leading to false groupings. However, the fact that a similar pattern is found with a different bead array assay from a second vendor (Immucor) in an independent population strongly argues against the impact of the bead manufacturing process and the importance of denatured antigens for this finding. Furthermore, stratification of the HLA-C response according to the HLA-C genetic background of the host showed that self-directed antibody responses are rare (**Supplementary Figure 2**). Indeed, antibody responses against denatured HLA molecules are one of the major causes of self-directed anti-HLA responses and are usually directed against a limited number of epitopes which are common between different HLA-A, -B, -C antigens and HLA–E molecules (34).

Since HLA-C alleles are inhibitory ligands protecting cellular targets from NK cell lysis, antibody responses against C1/NK2 and C2/NK1 alleles may play an important role in NK cell function in the transplantation context. Although the presence of C1 and C2 alleles is part of algorithms used for better allocation of bone marrow donors, the role of NK cells in organ transplantation is not studied so much (35, 36). However, very recent studies underlined a prominent role of NK1 *versus* NK2 responses for graft tolerance (32). It would be interesting to analyze the effect of anti-HLA-C antibody responses as a possible NK1 *versus* NK2 balance modifying factor.

Finally, we used a second more simple algorithmic approach to the whole reactions leading to a phylogenetic tree representation based on a distance matrix. Probably the most useful property of this representation is that it provides a simple way to define roots of main branches from where “speciation like” divergences emerge. It seems that a common antigenic structure forms the root of the branch from where subsequent sequence variations induce longer antigenic distances. Another interesting feature of this procedure resulting in a “species-like” projection of anti-HLA responses is the fact that these projections come from real data provided by Luminex platforms which are the main devices used to study anti-HLA responses nowadays. To this aspect, it parallels previous experimental methods used to define CREGs but can be used in existing data coming for numerous samples at no cost. In a certain way, the method provides a robust procedure to illustrate bead-array defined CREGs and then go into subsequent branches of the tree to define triplets, eplets or important physicochemical differences that form the basis of current algorithms used for the prediction of adverse immune responses against the graft.

The dendrogram of the antibody responses produced with this algorithmic approach put almost all the alleles of the same locus to the same branch of a tree. However, some distinct exceptions to this rule are illustrated. Anti-HLA-A*25:01, A*32:01 responses clustered among anti-HLA-Bw4 specific responses. A similar pattern was observed for anti-HLA-A*23:01, A*24:02, and A*24:03 responses that clustered in a branch containing Bw4 alleles (B*44:02, B*44:03) and Bw6 alleles (B*45:01 and B*15:12) whatever the distance measure (Minkowski or Manhattan, data not shown) used for the computation. These HLA-A alleles belonged to the Bw4 CREG and interestingly they were not well represented on the corresponding HLA-A PCA biplot, due probably to the strong influence of the response to shared polymorphic regions between HLA-Bw4 and HLA-A alleles. Respectively anti-HLA-B*46:01 responses clustered with anti HLA-C*03:02, C*03:03, C*03:04, C*07:02, C*12:03, C*01:02, C*16:01, C*14:02, and C*08:01 (C1 group), possibly indicating strong influence of the response to “shared” polymorphic regions between HLA-B46 (Bw6 allele) and C1 group.

However, one could argue that these speciation-like representations could be simply due to the presence of antibodies against denatured HLA which are in different proportions among different sets of bead arrays and produce groupings of reactions not related to epitope recognition. To investigate this possibility, we compared the dendrograms with the exact same settings for the HLA-A locus where the antigens presented on an OL, and an Immucor bead array was the most common, and the resulting tanglegram showed that the groupings of the corresponding antigens were very similar (**Supplementary Figure 3**). Moreover, a tanglegram of HLA-B and HLA-C responses in both dendrograms still classifies0 the B*46:01 allele within the HLA-C alleles although the B*46:01 allele is very rare in the Greek population. These findings further suggest that the proportion of denatured HLA antigens bound on the bead arrays does not influence clusters of the observable antigenic proximities.

Interestingly, neighbor-joining phylogenetic trees of gene region sequences of HLA-C genes show major differences with the antigenic distances shown by the immune response based dendrograms (37).

One of the limitations of our approach is that sera used in the study were considered positive either after a screening test or previous information of anti-HLA class I sensitization. This limits the conclusions that are only applicable to a population of pre/post-transplant patients. Moreover, one could argue that bead-arrays contain a proportion of denatured HLA molecules that tend to be recognized even in non-sensitized healthy individuals albeit at low levels. The presence of antibodies against denatured HLA which are in different proportions among different sets of bead arrays could produce groupings of reactions not related to epitope recognition. To investigate this possibility, concerning PCA projections, a similar approach in another smaller cohort selected on the same criteria and analyzed on an Immucor bead array showed a similar PCA pattern for anti-HLA-A, -B and -C responses (**Supplementary Figure 1**). The bead-arrays from Immucor and OL contain almost the same alleles, but the proportion of denatured antigens is not the same. Moreover, we compared dendrograms with the exact same settings for the HLA-A locus where the antigens present on an OL and an Immucor bead array were the most common, and the resulting tanglegram showed that the groupings of the corresponding antigens were very similar (**Supplementary Figure 3**). Moreover, a tanglegram of HLA-B and HLA-C responses in both dendrograms still classifies the B*46:01 allele within the HLA-C alleles although the B*46:01 allele is very rare in the Greek population. These findings further suggest that the proportion of denatured HLA antigens bound on the bead arrays does not influence clusters of the observable antigenic proximities.

Overall, we suggest that unsupervised modeling of the immune response as measured by Luminex platforms gives valuable information regarding antigenic distances among HLA alleles, and it is relatively easy to obtain from data stored in recent years in histocompatibility laboratories. Indeed, the use of PCA calculated angles and/or measures of phylogenetic distances among antigens could be another complementary approach used as a “weighing tool” for the improvement of predictions based on eplet load differences, EpMS, and PIRCHE scores. However, we recognize that more studies in different populations are necessary to provide more insights and support this mixed numerical and experimental approach.

## Data Availability Statement

The raw data supporting the conclusions of this article will be made available by the authors, without undue reservation.

## Ethics Statement

The studies involving human participants were reviewed and approved by the Health Research and Ethical Boards of Athens Gennimatas Hospital (31563/19.10.17), Thessaloniki Hippokrateion Hospital (291/21.02.2020) and Athens Evangelismos Hospital (46/26-02-2020). The patients/participants provided their written informed consent to participate in this study.

## Author Contributions

AV and AF: These authors have contributed equally to this work and share first authorship. AI and IT: These authors have contributed equally to this work and share last authorship. AV, AF, KT, ZT, AS, and GP: Performed the experiments, contributed to the design of statistical analyses, participated to the writing, and approved the final version. DG: Contributed to the conception of the statistical workflow, participated to the writing and approved the final version. CL, ID, and MC: Contributed to the design of the study, participated to the writing and approved the final version. AI and IT: Contributed to the design of the study and wrote the manuscript. All authors contributed to the article and approved the submitted version.

## Funding

The Hellenic Society for Immunology (HSI Ref: 9/29.01.2019).

## Conflict of Interest

The authors declare that the research was conducted in the absence of any commercial or financial relationships that could be construed as a potential conflict of interest.

## Publisher’s Note

All claims expressed in this article are solely those of the authors and do not necessarily represent those of their affiliated organizations, or those of the publisher, the editors and the reviewers. Any product that may be evaluated in this article, or claim that may be made by its manufacturer, is not guaranteed or endorsed by the publisher.

## Abbreviations

aa, amino acid; CREG, Cross Reactive Epitope Groups; Dim, dimension; DSA, donor specific antibodies; EDTA, ethylenediaminetetraacetic acid; EpMS, eplet mismatch score; HLA, human leukocyte antigen; LSM, LABScreen mixed; MFI, mean fluorescence intensity; OL, One Lambda; PCA, principal component analysis; PIRCHE, Predicted Indirectly Re-Cognizable HLA Epitopes; SAB, single antigen beads; TOT, total.
