# Supplementary material for: Hidden Patterns of Anti-HLA Class I Alloreactivity Revealed Through Machine Learning
Source: Front Immunol. 2021 Jul 27;12:670956. doi: 10.3389/fimmu.2021.670956 (PMC8353326; doi:10.3389/fimmu.2021.670956)

Supplementary figure 1A, 1B, 1C. PCA biplot projections of the anti-HLA class I antibody responses with Immucor

PCA projections of 830 single patient sera analyzed on a LIFECODES bead array (Immucor) for anti-HLA-A (panel 1A), anti-HLA-B (panel 1B) and anti-HLA-C (panel 1C) antibodies.

The points represent projections of individual reactions and arrows the different anti-HLA class I responses according to the first and second principal components (referred to as Dim1 and Dim2 accordingly) of PCA. The color stripe on the right side exhibits the corresponding color vectors of explained variance, ranging from red color (indicating strong contribution on variance) to green (indicating weak contribution on variance).

Panel 1A. PCA biplot projections of the anti-HLA-A antibody response

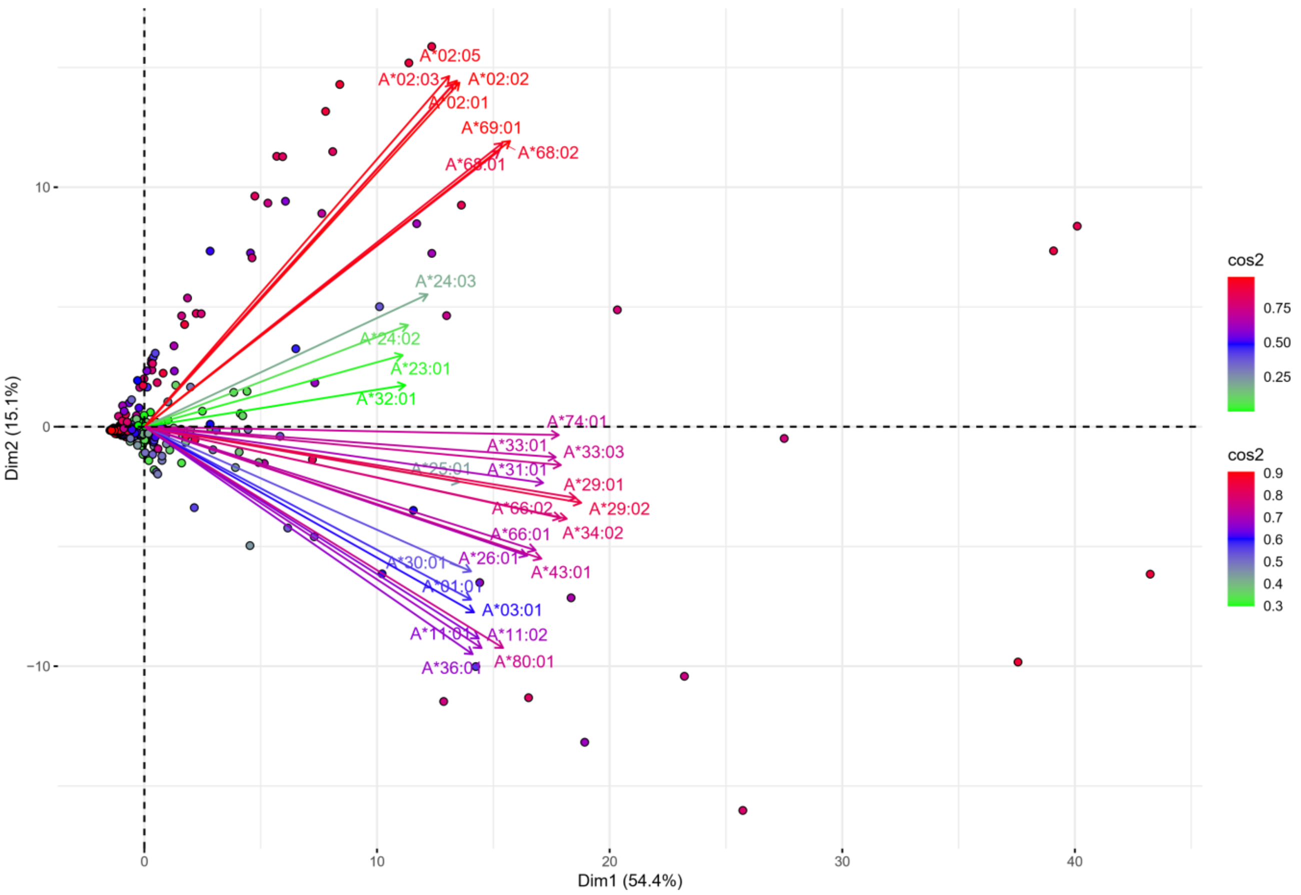

Panel 1B. PCA biplot projections of the anti-HLA-B antibody response

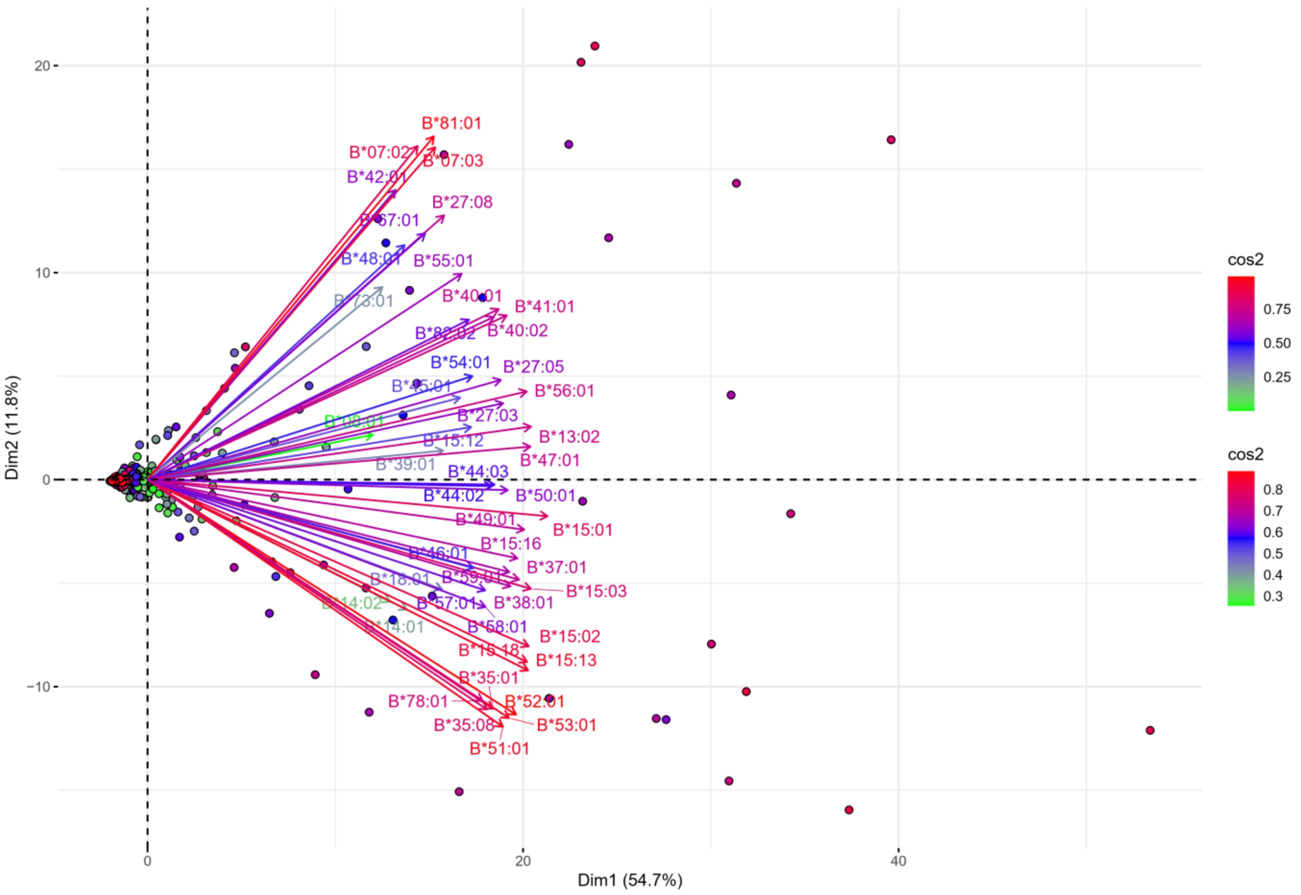

Panel 1C. PCA biplot projections of the anti-HLA-C antibody response

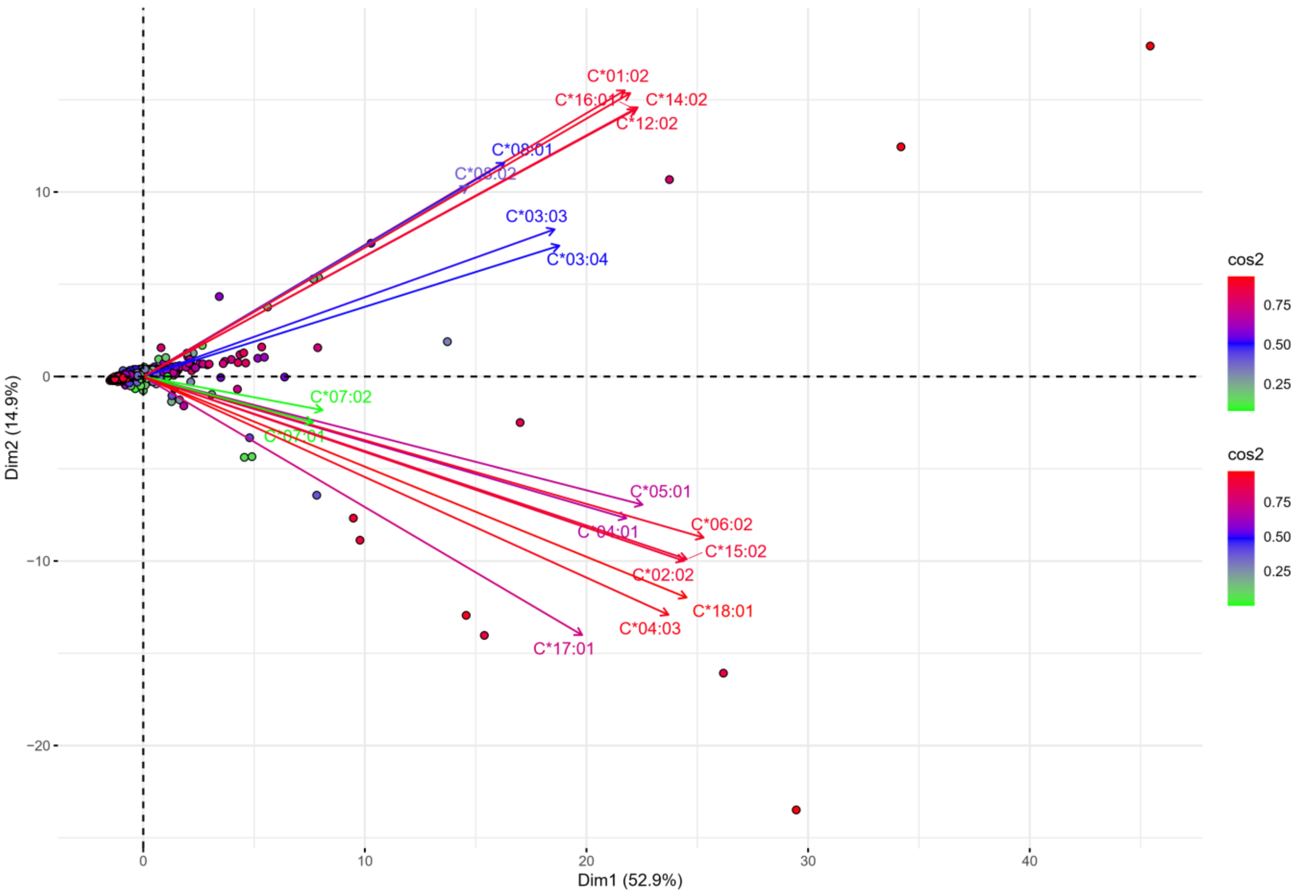

Supplement: Supplementary file 1 [file DataSheet_1.pdf]
