# Supplementary material for: Hidden Patterns of Anti-HLA Class I Alloreactivity Revealed Through Machine Learning
Source: Front Immunol. 2021 Jul 27;12:670956. doi: 10.3389/fimmu.2021.670956 (PMC8353326; doi:10.3389/fimmu.2021.670956)

Supplementary Figure 2. PCA biplots of anti HLA-C immune responses according to patient's HLA-C genetic background

Individual responses are colored red when the patient is positive for Cw4 (panel 2A) or Cw12 (panel 2B) genotypes and blue when negative. An ellipse containing 95% of individual responses is drawn around each group. These PCA projections derive from a subset of 1030 patients of the original cohort for which HLA-C molecular typing was available and include analyses for the two most frequent Cw4 and Cw12 alleles found in the cohort.

Panel 2A. Cw4 stratified PCA projections of HLA-C responses.

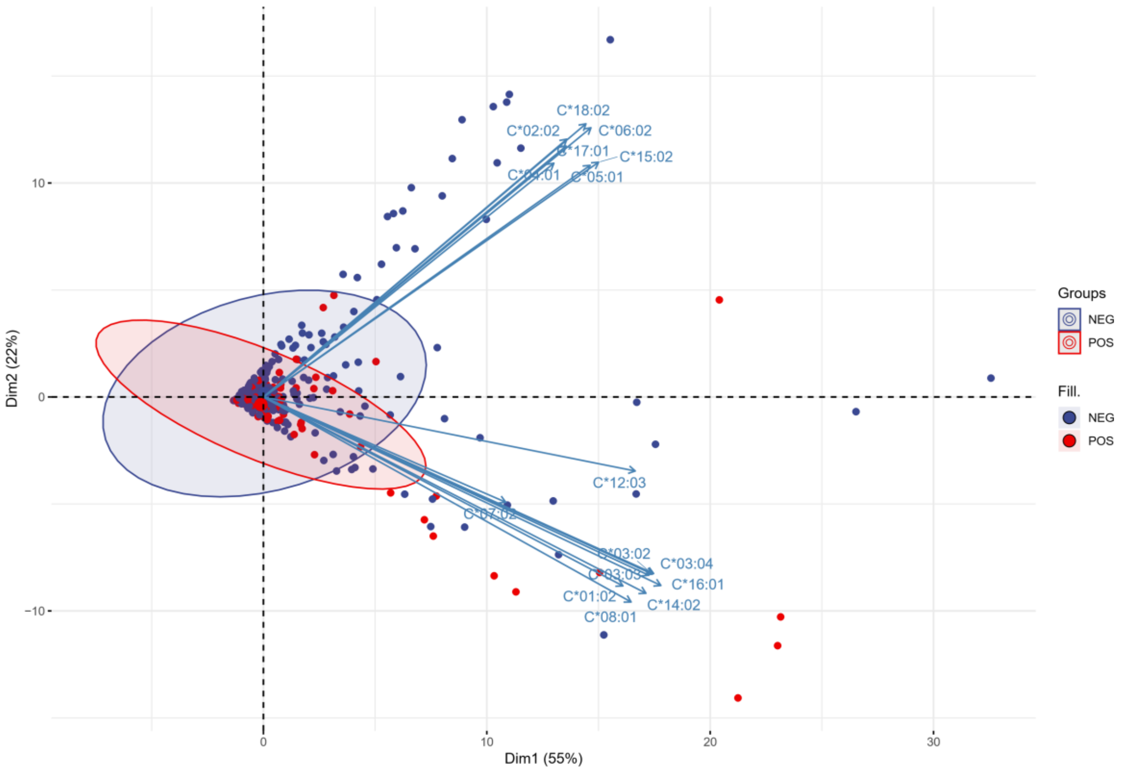

Panel 2B. Cw12 stratified PCA projections of HLA-C responses.

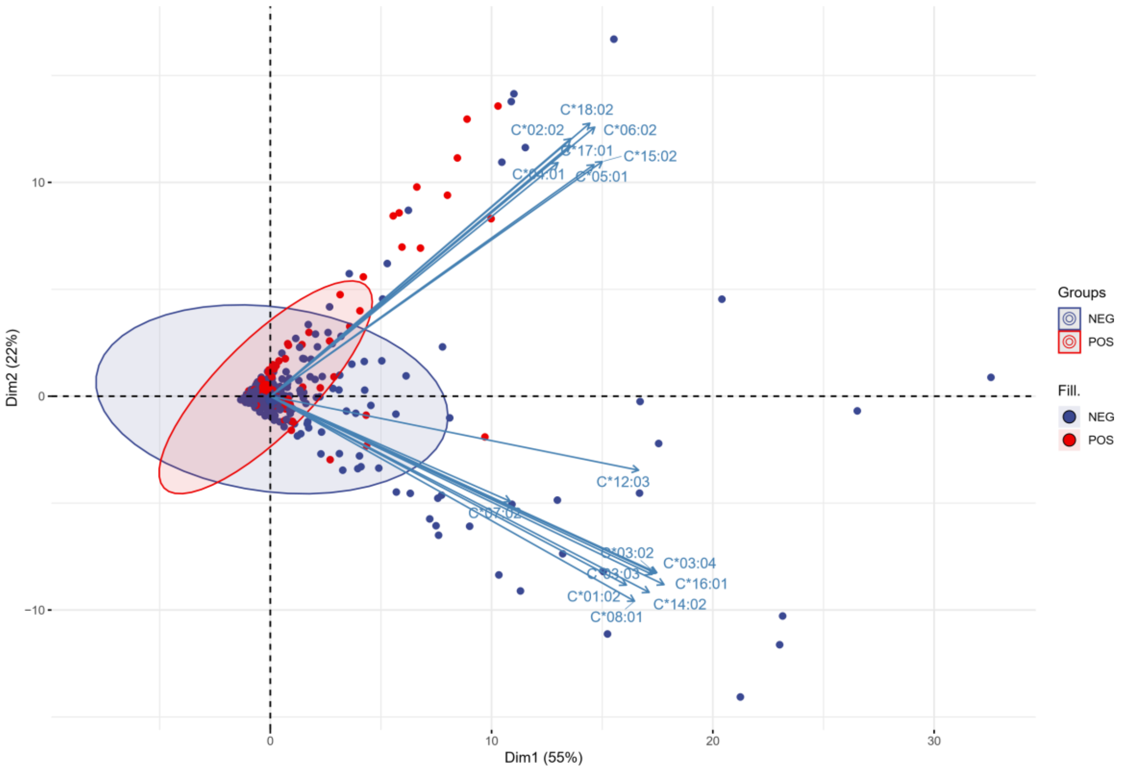

Supplement: Supplementary file 2 [file DataSheet_2.pdf]
