# Supplementary material for: Hidden Patterns of Anti-HLA Class I Alloreactivity Revealed Through Machine Learning
Source: Front Immunol. 2021 Jul 27;12:670956. doi: 10.3389/fimmu.2021.670956 (PMC8353326; doi:10.3389/fimmu.2021.670956)

Supplementary Fig.3. Dendrograms' comparison of anti HLA-A responses produced on an Immucor and One Lambda bead array.

The tanglegram compares feature vector dendrograms produced from a cohort of 830 patients analyzed with an Immucor bead array (left side) and a cohort of 1066 patients analyzed with an OL bead array (right side). Dendrograms were produced with the same algorithmic settings for the two different bead arrays. Only beads coated with the same antigens among the two types of reagents are represented.

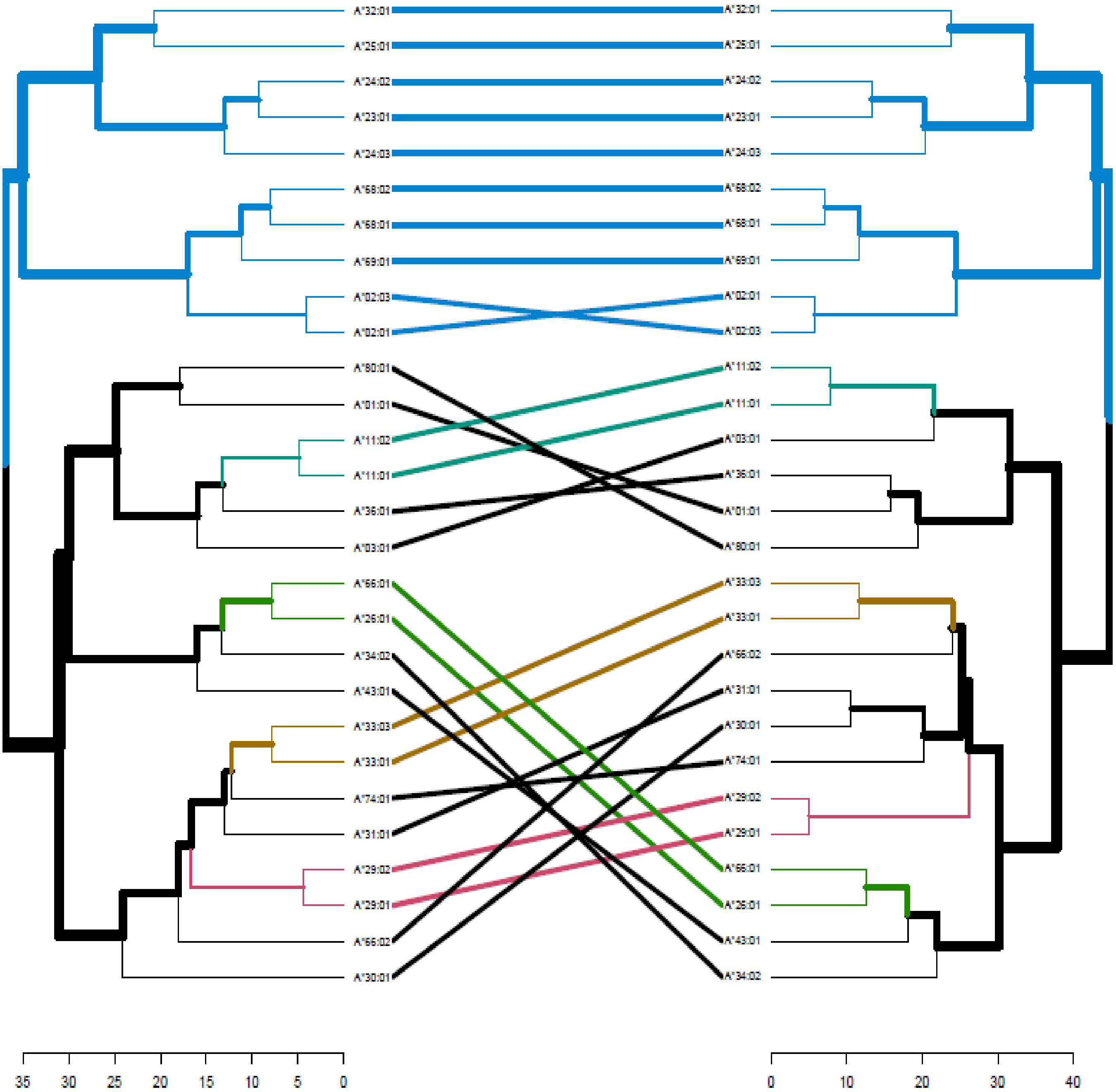

Supplement: Supplementary file 3 [file DataSheet_3.pdf]
